# Health belief model and social media engagement: A cross-national study of health promotion strategies against COVID-19 in 2020

**DOI:** 10.3389/fpubh.2023.1093648

**Published:** 2023-02-08

**Authors:** Zhifei Mao, Di Wang, Shanshan Zheng

**Affiliations:** ^1^School of Media and Communication, Shenzhen University, Shenzhen, China; ^2^Faculty of Humanities and Arts, Macau University of Science and Technology, Macau, China; ^3^Research Center for Pandemic and Epidemic Prediction and Intelligence, Faculty of Innovative Engineering, Macau University of Science and Technology, Macau, China

**Keywords:** COVID-19, public health, social media, online behavior, content analysis

## Abstract

**Background:**

Using the Health Belief Model (HBM), this study analyzed tweets related to COVID-19 published by national health departments of the United States, the South Korea, the United Kingdom, Japan, Germany, and India to explore their differences in (1) the health measures against COVID-19, (2) the health promotion strategies, (3) the social media engagements that those measures and strategies have triggered.

**Method:**

We conducted a content analysis with 1,200 randomly selected COVID-19-related tweets from six national health departments' Twitter accounts from 1 January 2020 to 31 December 2020. We coded the six HBM constructs and 21 sub-themes of the HBM constructs for each tweet.

**Results:**

Results showed that all six HBM constructs were used in the full sample. The most commonly used HBM construct was cues to action, followed by susceptibility, benefits, self-efficacy, severity, and barriers. All the HBM constructs were positively related to Twitter engagement variables except barriers. Further analysis illustrated that people from the six countries responded differently to the HBM constructs and the HBM sub-themes. Twitter users in Germany, India, the U.S., and Japan positively reacted to the clear directions of “what to do against COVID-19” (cues to action), while Twitter users in the U.S. and Japan were also eager to know the justifications for such directions (benefits); people in South Korea and the U.K. were mainly seeking a diagnosis of the severity and susceptibility of COVID-19, instead of health measures, of COVID-19 in the year 2020.

**Conclusions:**

This study showed the use of HBM constructs is generally effective in inducing Twitter engagement. The further comparison illustrated a homogenization in the promotion strategies that the health departments implemented and the health measures they promoted, yet responses to such promotions varied across nations. This study broadened the scope of HBM applications from predicting health behaviors in surveys to guiding the design of health promotion messages online.

## Introduction

Since the outbreak of COVID-19, policymakers all around the world have faced a common problem: how to persuade their citizens to embrace health policies to counter the epidemic. Before the COVID-19 vaccinations were released, health policies on COVID-19 were mainly focused on non-pharmaceutical health measures, such as wearing masks, maintaining social distancing, and personal sanitation (1). Previous studies have indicated that some of those policies, such as wearing masks and social distancing measures, were controversial and highly politicalized by various individuals and parties across different nations (2). Other policies or official suggestions, such as hygiene and sanitation measures such as washing hands, were much less debatable, yet were easily neglected or underestimated by citizens (3). Under these circumstances, it is crucial for policymakers to not only launch health policies related to COVID-19 swiftly but also to promote them to the public, convincing their citizens of the benefits of following the health policies.

Deviating from traditional studies that examined the media end and the audience end separately, data from the new media platforms granted us a chance to explore the media content and its corresponding effect simultaneously. We can analyze, for example, a particular tweet on COVID-19-related health policies and how Twitter users respond to it by measuring its engagement variables. By doing so, we can analyze the public promotion of health policies concerning COVID-19 from both the audiences' and the promotion strategies' end.

In this research, we borrow insights from the Health Belief Model (HBM) to conceptualize those strategies. The model illustrates that people's adoption of health behaviors is affected by several beliefs, including (a) perceived susceptibility (whether they are vulnerable to a disease), (b) perceived severity (the severity of a disease), (c) perceived barriers (the difficulty of preventative actions), (d) perceived benefits (the benefits of taking those actions), (e) self-efficacy (whether they can successfully implement the recommended health behavior), and (f) cues to actions (stimulus cues that trigger individuals to engage in appropriate health behaviors) (4, 5).

The classical way to apply HBM is through surveys, examining the relationship between people's beliefs and their health behaviors (6). Some researchers replicated this form of study to examine people's health beliefs related to COVID-19 (7–9). In recent years, new media have become an extremely important space for health promotion. Thus, only using the method of a survey to study HBM would lose valuable and rich information on social media. In addition, in surveys, people could be influenced by social desirability bias, and answer questions in a socially desirable way. But due to the anonymity of the Internet, people's likes and retweets in social media could more realistically reflect their true attitudes and interest. Therefore, the data on social media serve as an excellent platform to the study the effect of health promotion strategies.

Indeed, some studies have already explored HBM constructs on social platforms like Twitter. Such studies could be categorized into two categories. First, some researchers regarded HBM constructs as *the perceptions or attitudes of the public on a health crisis and its measures* (10–12). After the outbreak of COVID-19, some researchers used HBM constructs to identify people's perceptions of COVID-19 and its health measures, and by examining the frequency of which they could estimate to what extent people have formed a health belief of COVID-19 (13). These studies demonstrated the possibility of applying HBM to health promotion online, while failed to test the correlations between the health promotion strategies used by the health department and people's responses to them since they focused on the side of the public perceptions only. Others regarded HBM constructs as *different promotion strategies implemented by a health department*. For example, by examining the frequency of the constructs that appeared in health departments' Twitter accounts and people's reactions to those constructs, researchers could draw a picture of the preference of strategies that a health department would use to promote COVID-19 related health measures, as well as the effects of them (14, 15).

This study borrows the insights of the second group of studies to examine the correlations between the health promotion strategies of the health department (HBM constructs) and people's responses to them (likes and retweets). We will test whether the existence of the HBM constructs could induce higher Twitter engagements (likes and retweets). Furthermore, our contribution to the literature is 3-fold: First, this study shall take an in-depth look at the sub-themes of the HBM constructs to examine the *specific* health promotion strategies. Previous research usually treated each HBM construct as a whole without distinguishing the sub-themes of each construct. However, people may respond differently toward different health measures (e.g., they may like cues to action on vaccines but dislike or feel aloof for cues to action on masks). Therefore, it is imperative to examine the sub-themes within each HBM construct. Second, this study shall comprehensively profile Twitter users from different countries based on their different reactions toward the health promotion related to COVID-19 online. This helps the policymakers understand their citizens, and therefore they can further improve their communication strategies. Third, this study shall launch a cross-national comparative study to examine the similarities and differences between the health departments in their health promotion and the Twitter users in their responses to the health departments. Vermandere et al. (16) argued that it is necessary to test the application of health behavior theory in different environments to justify its rationality in promoting and intervening in health behavior in different settings. Previous studies have shown that the impact of the HBM constructs on Twitter engagement differed among the three major news agencies: the COVID-19 vaccination promotion using HBM constructs was effective for Reuters, but seems to be counterproductive for AFP (17). Moreover, people in different societies also have their favored ways of regarding Twitter engagements (18). Taking the possible differences among societies into consideration, we will implement a comparative study to answer the following questions:

RQ1: What are the differences in using the HBM constructs between the six national health departments' tweets?
RQ2: Does Twitter engagement vary across the six national health departments?
RQ3: To what extent does the presence of HBM constructs in tweets by national health departments impact Twitter engagement?
RQ4: Does the effect of HBM constructs on Twitter engagement vary across the six national health departments?

## Method

We used Python^®^ to retrieve information about COVID-19 on Twitter using Twitter's Application Programming Interfaces in January 2021. The time frame of the study was from January 1, 2020, to December 31, 2020. The year 2020 was selected because 2020 was the first year of the COVID-19 outbreak. For most countries, COVID-19 vaccines were not available until December 2020. Before the advent of the COVID-19 vaccines, countries around the world implemented many non-pharmaceutical measures, such as social distancing, wearing masks, and even lockdowns. However, after the advent of the COVID-19 vaccine, many measures, such as lockdowns, were canceled (1). Therefore, 2020 is of great significance for health departments across the globe to promote health measures to fight against COVID-19.

A quantitative content analysis was conducted with 1,200 randomly selected COVID-19-related tweets from six national health departments' Twitter accounts in 2020. To control the impact of the economy on policies, the six countries were selected based on the rank of Gross Domestic Product (GDP) in 2020 (19). To further diversify our data, we drew the three highest-ranking GDP countries from the East (Japan, India, and South Korea) and the West (the U.S., Germany, and the U.K.). China, the country with the largest GDP in the East, was excluded from the sample because Twitter is not available there.

The search key words were “2019nCoV, 2019-nCoV, 2019n_CoV, Coronavirus, Corona, Novel coronavirus, novelcoronavirus2019, COVID, COVID19, COVID-19, COVID2019, nCoV2019, NCOV19, NCOV, nCoV2020, neuartige virus, virus, Lungenentzündung, 新型コロナウイルス,コロナウイルス, コロナ,ウイルス,肺炎,코로나19, 바이러스, 신종코로나바이러스, 코로나 바이러스, 코로나, 바이러스, 코로나바이러스감염증. ” As COVID-19 was named “不明肺炎 ”, or unknown pneumonia in Japan before the WHO named it COVID-19 on Feb 11, 2020, we included “肺炎 ” in the Japanese search words. Tweets about pneumonia, but was not related to COVID-19, were excluded manually later. After manually excluding unrelated tweets, a total of 15,856 tweets were downloaded, including 1,558 tweets about COVID-19 from the U.S. Department of Health and Human Services' Twitter account (@HHSGov), 2,027 tweets from the Germany Federal Ministry of Health's Twitter account (@BMG_Bund), 2,602 tweets from the Japanese Ministry of Health, Labour and Welfare's Twitter account (@MHLWitter), 3,302 tweets from the U.K. Department of Health and Social Care's Twitter account (@DHSCgovuk), 2,375 tweets from the India Ministry of Health's Twitter account (@MoHFW_INDIA), and 3,992 tweets from the South Korea Ministry of Health and Welfare's Twitter account (@mohwpr).

We randomly selected 200 tweets from the search results of each of the six national health departments' Twitter accounts. The full text, as well as images and videos (the length of the video ranged from 1 to 55 min) of the 1,200 tweets, were examined. All the files were downloaded in English except those from Germany, Japan, and South Korea. Videos were converted into audio files and then to text by using professional audio conversion software, Xunjie^®^. Then all the text was translated into English by professional translators for analysis. The data for Twitter-specific variables were downloaded from the Twitter website including the number of likes, and the number of retweets. Each tweet was examined for one or more of the HBM constructs, that is, when the six HBM constructs are coded, it is a multiple choice rather than a single choice. We also coded 21 sub-themes of the HBM constructs. Following Glanz (20) and the health policies of each country (9), the operationalizations of the HBM variables are shown in Table 1.

**Table 1:** Operationalization of HBM variables.

**HBM constructs and operationalization**	**Sub-themes**	**Examples**
1. Susceptibility [define population(s) at risk, and risk levels; personalize risk based on a person's features or behavior; heighten susceptibility if too low.]	1.1. The susceptibility of COVID-19 to the vulnerable group	Some people are at higher risk of being hospitalized if they get #coronavirus. Read NHS advice which sets out very clearly the different advice to different groups who are more vulnerable to #COVID19.
	1.2. The susceptibility of COVID-19 to the general public	The number of people infected with the new corona is increasing rapidly in some areas.
2. Severity (specify the consequences of the risk and the condition.)	2.1. The severity of COVID-19 to the vulnerable group	Older adults and people who have severe chronic medical conditions like heart, lung, or kidney disease or diabetes, may be at higher risk for severe illness from COVID-19.
	2.2. The severity of COVID-19 to the general public	As of 9 am 30 March, a total of 134,946 have been tested: 112,805 negative. 22,141 positive. As of 5 p.m. on 29 March, of those hospitalized in the U.K., 1,408 have sadly died.
3. Benefits (clarify the positive effects of taking the advised action to reduce the risk or seriousness of the impact.)	3.1. Benefits of physical and social distancing measures for the general public	Wearing a face covering and staying six feet apart doesn't just protect you, it protects those around you.
	3.2. Benefits of personal measures	A mask is one of the best ways to help prevent the spread of COVID-19.
	3.3. Benefits of virus testing and patient tracking	Testing is free, quick, and vital to stop the spread of coronavirus.
	3.4. Benefits of pharmaceutical interventions	An effective vaccine is the biggest breakthrough since #COVID19 was identified. It could save thousands of lives. Learn more about #COVID19 vaccination: http://nhs.uk/covidvaccine
4. Barriers (identify the tangible and psychological costs of the advised action.)	4.1. Barriers to medical resources strategies	This means that hospitals, medical practices, and nursing homes hardly have a chance to replenish their stocks and procure what they need in such a highly competitive market.
5. Cues to action (remind to take action.)	5.1. Cues to action on movement restriction	Several areas in England are moving into higher tiers from 00:01 tomorrow. 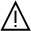 This is to limit the spread of #COVID19 as cases continue to rise across the country. See the list of local restriction tiers by area
	5.2. Cues to action on physical and social distancing measures for confirmed/suspected cases	If you have symptoms of COVID-19 (new continuous cough OR a high temperature), it's important that you stay at home for 7 days to help protect your friends and neighbors.
	5.3. Cues to action on physical and social distancing measures for the general public	Have plans this weekend? If you will be around others, stay at least 6 ft apart and wear a cloth face covering to slow the spread of #COVID19.
	5.4. Cues to action on personal measures	Continue social distancing, wearing a face covering, and washing your hands frequently to help protect yourself and others around you from #COVID19.
	5.5. Cues to action on the protection of special groups	Before COVID-19 vaccines are authorized, a CDC advisory committee recommended healthcare personnel and long-term care facility residents should receive #COVID19 vaccination first, while supplies are limited.
	5.6. Cues to action on medical resources strategies	We recently announced that 15,000 @PenlonGlobal devices will be sent to the #NHS frontline to support coronavirus (#COVID19) patients.
	5.7. Cues to action on virus testing and patient tracking	Are you 65 or over and live in England? If you or anyone in your household has #coronavirus symptoms, you can book a test online.
	5.8. Cues to action on pharmaceutical interventions	“I want to encourage everyone who has the opportunity, to get vaccinated so that we can have a veil of protection over this country that would end this pandemic.” - Dr. Anthony Fauci
6. Self-efficacy (provide training and guidance in performing an action to increase people's self-efficacy in dealing with COVID-19.)	6.1. Self-efficacy (training or guidance on physical and social distancing measures for confirmed/suspected cases)	You can take a medical examination, check your fever, and take a sample through the car window while in the car. By minimizing contact between medical staff and patients, the risk of infection can be reduced and the speed of testing can be increased.
	6.2. Self-efficacy (training or guidance on personal measures)	Wash your hands more often. Use soap and water for 20 s or use hand sanitizer.
	6.3. Self-efficacy (training or guidance on virus testing and patient tracking)	Does your child need to have a #COVID19 test? If you're taking your child for a test, show them this video to explain what will happen 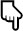 If your child has any #coronavirus symptoms, book a test 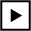 call 119 or visit http://NHS.uk
	6.4. Self-efficacy (training or guidance on pharmaceutical interventions)	General Gus Perna, Chief Operating Officer for Operation Warp Speed (#OWS), explains the five key tenets behind the successful operation to rapidly develop, produce and distribute a safe and effective #COVID19 vaccine to the American people.

Two graduate students who are fluent in English coded all the files. We calculated the inter-coder reliability of the two coders by double-coding a random subsample (*n* = 240 or 20%) of the data. Krippendorff's alpha ranged from 0.85 to 1.0 for the six main variables (susceptibility, severity, benefits, barriers, cues to action, and self-efficacy) and 0.80–1.0 for the 21 sub-themes.

## Results

### HBM constructs used in six national health department's tweets

To answer RQ1 and RQ2, we will use Chi-square tests to examine the differences in the HBM constructs and Twitter engagement among the six national health departments' tweets. As can be seen in Table 2, the most often used HBM construct was cues to action (*n* = 490, 40.8%), followed by susceptibility (*n* = 74, 6.2%), benefits (*n* = 56, 4.7%), self-efficacy (*n* = 30, 2.5%), severity (*n* = 28, 2.3%), and barriers (*n* = 4,0.3%).

**Table 2:** The frequency of HBM constructs used by country.

	**Susceptibility**	**Severity**	**Benefits**	**Barriers**	**Cues to action**	**Self-efficacy**
Total	74 (6.2)	28 (2.3)	56 (4.7)	4 (0.3)	490 (40.8)	30 (2.5)
The U.K.	6 (3.0)	2 (1.0)	8 (4.0)	1 (0.5)	106 (53.0)	8 (4.0)
The U.S.	13 (6.5)	2 (1.0)	25 (12.5)	0 (0)	102 (51.0)	10 (5.0)
Germany	16 (8.0)	2 (1.0)	9 (4.5)	3 (1.5)	74 (37.0)	4 (2.0)
Japan	15 (7.5)	9 (4.5)	1 (0.5)	0 (0)	51 (25.5)	1 (0.5)
South Korea	10 (5.0)	5 (2.5)	7 (3.5)	0 (0)	84 (42.0)	2 (1.0)
India	14 (7.0)	8 (4.0)	6 (3.0)	0 (0)	73 (36.5)	5 (2.5)
Chi-square	5.99	11.26^*^	37.46^***^	11.04	43.16^***^	12.31^*^

Values inside the parenthesis represent the percentage of *n*.

* *p* < 0.05;

*** *p* < 0.001.

**Figure 1:**
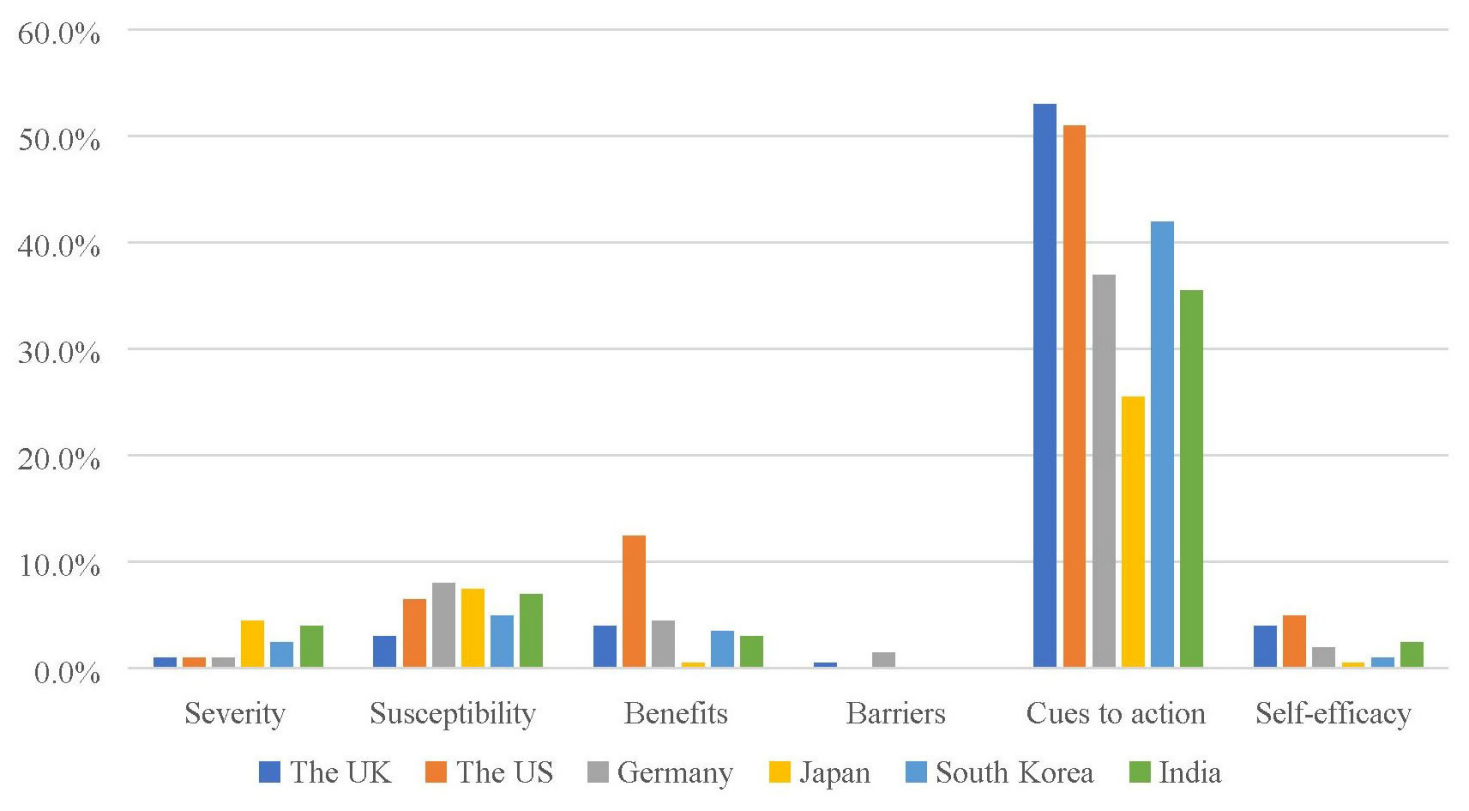
HBM constructs used by the six national health department's tweets.

When we compare the use of each HBM construct by country, results showed that the six national health department's Twitter accounts showed significant difference in the frequency of severity (χ^2^ = 11.26, *p* < 0.05), benefits (χ^2^ = 37.41, *p* < 0.001), cues to action (χ^2^ = 43.16, *p* < 0.001), and self-efficacy (χ^2^ = 12.31, *p* < 0.05). *Post-hoc* analysis showed that the U.S. health department mentioned significantly more the benefits of taking preventive behaviors (*n* = 25) than the expected count (*n* = 9.3) while the Japanese health department mentioned significantly fewer benefits of taking preventive behaviors (*n* = 1) than the expected count (*n* = 9.3). The U.K. and the U.S. health department mentioned significantly more cues to action (*n* = 106 for the U.K. and *n* = 102 for the U.S.) in their tweets than the expected count (*n* = 81.7). For visual comparison, see Figure 1.

Table 3 showed the frequency of sub-themes of the HBM constructs by country. For the whole sample, the most mentioned sub-themes were those under cues to action, such as cues to action on personal measures (*n* = 174, 14.5%) and on physical and social distancing measures for the general public (*n* = 154, 12.8%). Sub-themes that were mentioned less frequently are those under susceptibility, such as the susceptibility of COVID-19 to the general public (*n* = 57, 4.8%). Sub-themes under severity, benefit, and self-efficacy were seldom mentioned, while the only sub-theme of barrier, barriers of medical resources strategies, was mentioned only four times (0.3%).

**Table 3:** The frequency of the sub-themes of the HBM constructs by country.

**Sub-themes of HBM constructs**	**UK**	**US**	**Germany**	**Japan**	**South Korea**	**India**	**Total**
1.1. The susceptibility of COVID-19 to the vulnerable group	1 (0.5)	6 (3.0)	8 (4.0)	0 (0)	1 (0.5)	3 (1.5)	19 (1.6)
1.2. The susceptibility of COVID-19 to the general public	5 (0.5)	7 (3.5)	9 (4.5)	16 (8.0)	9 (4.5)	11 (5.5)	57 (4.8)
2.1. The severity of COVID-19 to the vulnerable group	0 (0)	1 (0.5)	1 (0.5)	0 (0)	2 (1.0)	1 (0.5)	5 (0.4)
2.2. The severity of COVID-19 to the general public	2 (1.0)	1 (0.5)	1 (0.5)	9 (4.5)	2 (1.0)	7 (3.5)	22 (1.8)
3.1. Benefits of physical and social distancing measures for the general public	0 (0)	6 (3.0)	0 (0)	0 (0)	0 (0)	0 (0)	6 (0.5)
3.2. Benefits of personal measures	4 (2.0)	15 (7.5)	3 (1.5)	1 (0.5)	0 (0)	3 (1.5)	26 (2.2)
3.3. Benefits of virus testing and patient tracking	2 (1.0)	1 (0.5)	0 (0)	0 (0)	1 (0.5)	0 (0)	4 (0.3)
3.4. Benefits of pharmaceutical interventions	2 (1.0)	3 (1.5)	3 (1.5)	0 (0)	0 (0)	0 (0)	8 (0.7)
4.1. Barriers to medical resources strategies	1 (0.5)	0 (0)	3 (1.5)	0 (0)	0 (0)	0 (0)	4 (0.3)
5.1. Cues to action on movement restriction	2 (1.0)	2 (1.0)	3 (1.5)	0 (0)	10 (5.0)	0 (0)	17 (1.4)
5.2. Cues to action on physical and social distancing measures for confirmed/suspected cases	16 (8.0)	5 (2.5)	1 (0.5)	3 (1.5)	2 (1.0)	2 (1.0)	29 (2.4)
5.3. Cues to action on physical and social distancing measures for the general public	27 (13.5)	34 (17.0)	24 (12.0)	5 (2.5)	30 (15.0)	34 (17.0)	154 (12.8)
5.4. Cues to action on personal measures	26 (13.0)	49 (24.5)	29 (14.5)	16 (8.0)	14 (7.0)	40 (20.0)	174 (14.5)
5.5. Cues to action on the protection of special groups	0 (0)	2 (1.0)	1 (0.5)	0 (0)	2 (1.0)	3 (1.5)	8 (0.7)
5.6. Cues to action on medical resources strategies	2 (1.0)	5 (2.5)	8 (4.0)	0 (0)	9 (4.5)	3 (1.5)	27 (2.3)
5.7. Cues to action on virus testing and patient tracking	42 (21.0)	3 (1.5)	10 (5.0)	0 (0)	6 (3.0)	3 (1.5)	64 (5.3)
5.8. Cues to action on pharmaceutical interventions	4 (2.0)	15 (7.5)	7 (3.5)	0 (0)	0 (0)	5 (2.5)	31 (2.6)
6.1. Self-efficacy (training or guidance on physical and social distancing measures for confirmed/suspected cases)	0 (0)	0 (0)	0 (0)	1 (0.5)	0 (0)	0 (0)	1 (0.1)
6.2. Self-efficacy (training or guidance on personal measures)	7 (3.5)	9 (4.5)	4 (2.0)	1 (0.5)	2 (1.0)	5 (2.5)	28 (2.3)
6.3. Self-efficacy (training or guidance on virus testing and patient tracking)	1 (0.5)	0 (0)	0 (0)	0 (0)	0 (0)	0 (0)	1 (0.1)
6.4. Self-efficacy (training or guidance on pharmaceutical interventions)	0 (0)	1 (0.5)	0 (0)	0 (0)	0 (0)	0 (0)	1 (0.1)

Values inside the parenthesis represent percentage of *n*.

When we break the results by country, a similar pattern can be observed. One exception was Japan, which only emphasized the susceptibility and the severity of COVID-19 to the general public and a few sub-themes of cues to action.

### Differences in the Twitter engagement

**Figure 2:**
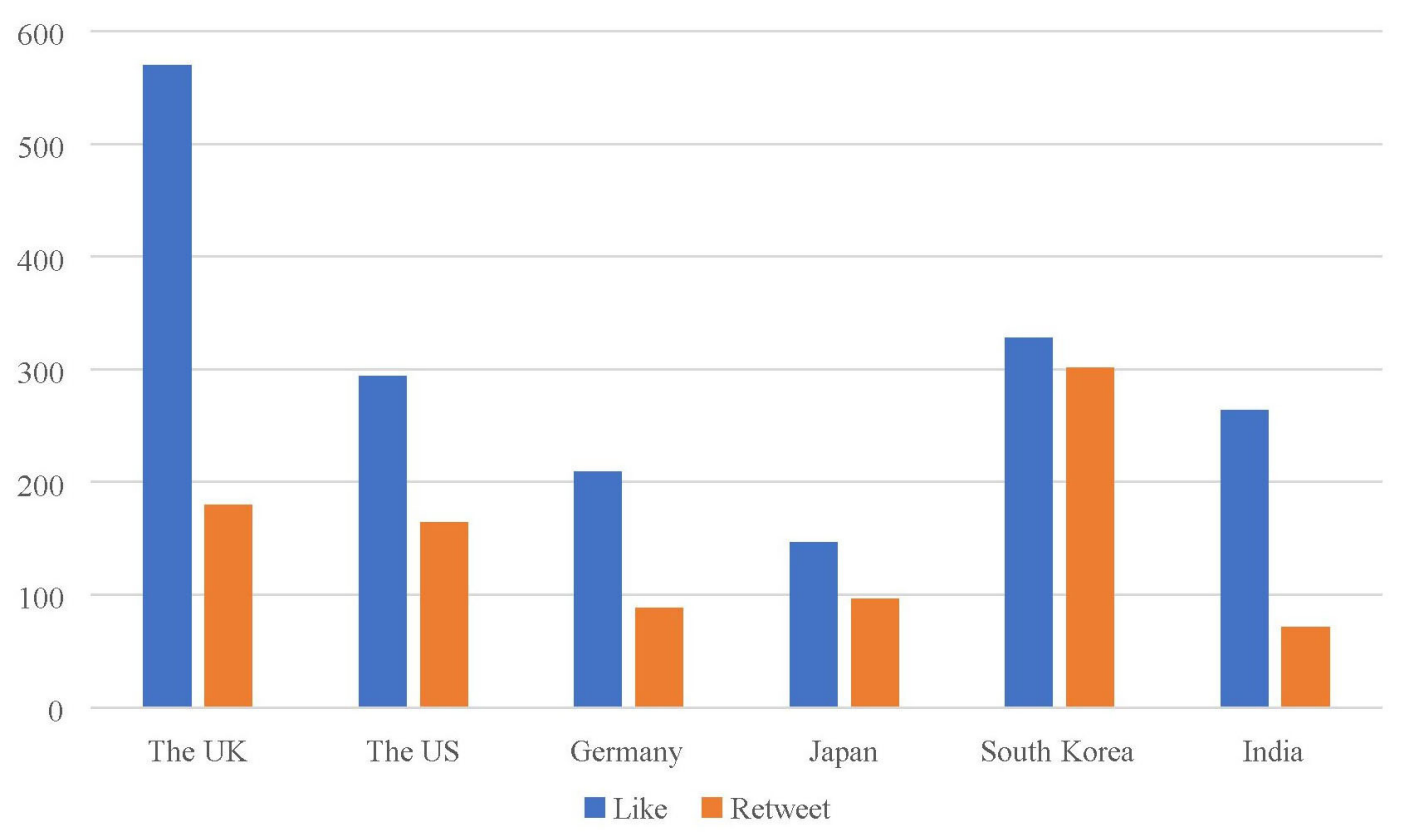
The mean number of likes and retweets by country.

For the entire sample, the mean number of retweets was 150.43 (*SD* = 516.79) and the mean number of likes was 302.43 (*SD* = 2,046.05). One-way ANOVA test showed that the six countries' health departments did not differ significantly in terms of the number of likes, *F*_(5,1,194)_ = 1.02, *p* > 0.05, but differed significantly in terms of the number of retweets, *F*_(5,1,194)_ = 5.63, *p* < 0.001. As our data met the assumption of homogeneity of variances, we used Tukey's honestly significant difference (HSD) *post-hoc* test to further test the differences in the number of retweets across countries. *Post-hoc* (HSD) analysis showed that South Korea's number of retweets (*M* = 301.36, *SD* = 751.86) was higher than that of Germany (*M* = 88.37, *SD* = 288.46), Japan (*M* = 96.48, *SD* = 256.65) and India (*M* = 71.57, *SD* = 297.04). All six countries' data showed a positive skew, which indicates that the tail is on the right side of the distribution. The kurtosis values were all >3, which meant that the data produced more outliers than the normal distribution (see Table 4). Figure 2 showed the visual comparison of Twitter engagement across countries.

**Table 4:** Descriptive statistics for Twitter engagement in the six national health departments' tweets.

**Country**	**Engagement variable**	**Range**	**Mean (SD)**	**Skewness**	**Kurtosis**	***N***
The U.K.	Like	14–64,644	570.18 (4,575.20)	13.94	196.17	200
	Retweet	3–11,186	180.25 (808.31)	12.87	196.17	200
The U.S.	Like	0–8,909	294.30 (805.23)	7.68	71.76	200
	Retweet	0–2,789	164.56 (340.84)	5.06	30.15	200
Germany	Like	0–8,267	209.38 (670.05)	9.60	108.79	200
	Retweet	0–2,967	88.37 (288.46)	7.27	61.35	200
Japan	Like	40–2,205	147.06 (238.64)	6.06	41.68	200
	Retweet	14–3,306	96.48 (256.65)	10.42	125.36	200
South Korea	Like	2–10,721	329.58 (1,133.64)	7.48	62.45	200
	Retweet	0–6,617	301.36 (751.86)	6.35	47.60	200
India	Like	6–18,729	264.11 (1,320.59)	13.88	194.91	200
	Retweet	0–4,219	71.57 (297.04)	13.82	193.80	200
Total	Like	0–64,644	302.43 (2,046.05)	26.94	823.71	1,200
	Retweet	0–11,186	150.43 (516.79)	12.74	218.42	1,200

### The effect of the HBM constructs in tweets on Twitter engagement

As the Twitter engagement variables were not normally distributed, we used non-parametric Mann-Whitney *U*-tests to examine the effect of the presence of HBM constructs on Twitter engagement, in response to RQ3 and RQ4. Mann Whitney *U*-test is used to compare the difference between two independent groups when the dependent variable is ordinal or continuous but not normally distributed and is generally considered a non-parametric alternative to the independent *t*-test (21). In our study, we compared the tweets that used the HBM constructs with those that did not use the HBM constructs.

Table 5 showed that tweets emphasizing the susceptibility of COVID-19 were liked more often (Mean ranks = 688.12) than tweets that did not emphasize the susceptibility of COVID-19 (Mean ranks = 594.74), Mann-Whitney *U* = 35,178.00, *p* < 0.05; tweets emphasizing the severity of COVID-19 were liked more often (Mean ranks = 761.59) than tweets that did not emphasize the severity of COVID-19 (Mean ranks = 596.65), Mann-Whitney *U* = 11,897.50, *p* < 0.05.

**Table 5:** HBM constructs and Twitter engagement.

**HBM construct**	**Engagement variables**	**Mean ranks of the group with the HBM variable present**	**Mean ranks of the group with the HBM variable absent**	**Mann-Whitney *U***	***Z***
Susceptibility	Retweets	673.85	595.68	36,234.00	−1.88
	Likes	688.12	594.74	35,178.00^*^	−2.25
Severity	Retweets	716.89	597.72	13,149.00	−1.80
	Likes	761.59	596.65	11,897.50^*^	−2.49
Benefits	Retweets	717.53	594.77	25,478.50^*^	−2.59
	Likes	713.91	594.95	25,681.00^*^	−2.51
Barriers	Retweets	578.13	600.57	2,302.50	−0.13
	Likes	675.38	600.25	2,092.50	−0.43
Cues to action	Retweets	677.13	547.62	136,402.00^***^	−6.36
	Likes	663.28	557.17	143,188.50^***^	−5.21
Self-efficacy	Retweets	772.33	596.09	12,395.00^**^	−2.75
	Likes	729.68	597.19	13,674.50^*^	−2.07

* *p* < 0.05;

** *p* < 0.01;

*** *p* < 0.001.

Tweets emphasizing the benefits of taking preventative measures were retweeted more often (Mean ranks = 717.53) than tweets that did not emphasize the benefits of taking preventative measures (Mean ranks = 594.77), Mann-Whitney *U* = 25,478.50, *p* < 0.05. Similarly, tweets emphasizing the benefits of taking preventative measures were also liked more often (Mean ranks = 713.91) than tweets that did not emphasize the benefits of taking preventative measures (Mean ranks = 594.95), Mann-Whitney *U* = 25,681.00, *p* < 0.05.

Tweets emphasizing cues to action were retweeted more often (Mean ranks = 677.13) than tweets that did not emphasize cues to action (Mean ranks = 547.62), Mann-Whitney *U* = 136,402.00, *p* < 0.001. Similarly, tweets emphasizing cues to action were also liked more often (Mean ranks = 663.28) than tweets that did not emphasize cues to action (Mean ranks = 557.17), Mann-Whitney *U* = 143,188.50, *p* < 0.001.

Tweets emphasizing training and guidance to increase self-efficacy were retweeted more often (Mean ranks = 772.33) than tweets that did not emphasize training and guidance to increase self-efficacy (Mean ranks = 596.09), Mann-Whitney *U* = 12,395.00, *p* < 0.01. Similarly, tweets emphasizing training and guidance to increase self-efficacy were also liked more often (Mean ranks = 729.68) than tweets that did not emphasize training and guidance to increase self-efficacy (Mean ranks = 597.19), Mann-Whitney *U* = 13,674.50, *p* < 0.05. The only HBM variable that did not have an impact on Twitter engagement is barriers.

To explore what specific topics were liked and/or retweeted most under the six HBM constructs, we also ran a series of Mann-Whitney *U*-tests to explore the relationship between the sub-themes of the HBM constructs and Twitter engagement for the whole sample. Table 6 showed that seven sub-themes of the HBM constructs [the severity of COVID-19 to the vulnerable group, benefits of physical and social distancing measures for the general public, benefits of personal measures, cues to action on movement restriction, cues to action on physical and social distancing measure for the general public, cues to action on personal measures and self-efficacy (training or guidance on personal measures)] were positively related to the Twitter engagement while one sub-theme (cues to action on pharmaceutical interventions) was negatively related to Twitter retweet for the whole sample.

**Table 6:** Sub-themes of the HBM constructs and Twitter engagement for the whole sample.

**Sub-theme of HBM constructs**	**Engagement variables**	**Mean ranks of the group with the HBM variable present**	**Mean ranks of the group with the HBM variable absent**	**Mann-Whitney *U***	***Z***
2.1. The severity of COVID-19 to the vulnerable group	Retweets	892.40	599.28	1,528.00	−1.89
	Likes	956.30	599.01	1,208.50^*^	−2.30
3.1. Benefits of physical and social distancing measures for the general public	Retweets	1,010.83	598.44	1,120.00^**^	−2.91
	Likes	1,026.50	598.36	1,026.00^**^	−3.02
3.2. Benefits of personal measures	Retweets	773.06	596.68	10,775.50^*^	−2.57
	Likes	760.10	596.97	11,112.50^*^	−2.37
5.1. Cues to action on movement restriction	Retweets	810.06	597.49	6,493.00^*^	−2.51
	Likes	681.82	599.33	8,673.00	−0.97
5.3. Cues to action on physical and social distancing measures for the general public	Retweets	738.13	578.41	62,976.00^***^	−5.51
	Likes	717.26	581.76	66,440.00^***^	−4.68
5.4. Cues to action on personal measures	Retweets	736.45	576.04	68,176.50^***^	−5.77
	Likes	738.91	575.59	677,726.50^***^	−5.87
5.8. Cues to action on pharmaceutical interventions	Retweets	436.33	604.57	12,218.50^*^	−2.59
	Likes	556.28	601.60	15,697.00	−0.70
6.2. Self-efficacy (training or guidance on personal measures)	Retweets	810.25	595.49	10,535.00^**^	−3.24
	Likes	769.75	596.46	11,669.00^**^	−2.62

* *p* < 0.05;

** *p* < 0.01;

*** *p* < 0.001.

To further understand the relationship between the HBM constructs and Twitter engagement for each country, we ran a series of Mann-Whitney *U-*tests to explore the relationship between the HBM constructs and Twitter engagement for by country. When we ran the analysis by country, we can see four of the HBM constructs (susceptibility, benefits, cues to action, and self-efficacy) were effective for the U.S., while only some of the HBM constructs were effective for the other five countries in inducing Twitter engagement (see Table 7). Specifically, severity was a positive predictor of the number of likes for the U.K.; cues to action was the only HBM that is positively related to Twitter engagement for Germany and India; severity and vulnerability were effective in inducing Twitter engagement for South Korea; benefits and cues to action are positive related to Twitter engagement for Japan.

**Table 7:** The HBM constructs and Twitter engagement by country.

	**HBM variable**	**Engagement variables**	**Mean ranks of the group with the HBM variable present**	**Mean ranks of the group with the HBM variable absent**	**Mann-Whitney *U***	***Z***
U.K.	Severity	Retweets	179.50	99.70	40.00	−1.94
		Likes	180.50	99.69	38.00^*^	−1.97
U.S.	Susceptibility	Retweets	143.81	97.49	652.50^**^	−2.79
		Likes	135.08	98.10	766.00^*^	−2.23
	Benefits	Retweets	135.16	95.55	1,321.00^**^	−3.20
		Likes	135.32	95.53	1,317.00^**^	−3.22
	Cues to action	Retweets	112.64	87.87	3,760^**^	−3.03
		Likes	115.21	85.19	3,498.00^***^	−3.67
	Self-efficacy	Retweets	154.95	97.63	405.50^**^	−3.05
		Likes	153.25	97.72	422.50^**^	−2.96
Germany	Cues to action	Retweets	114.68	92.17	3,613.00^**^	−2.66
		Likes	113.93	92.61	3,668.00^*^	−2.52
Japan	Benefits	Retweets	198.00	100.01	2.00^*^	−1.69
		Likes	197.00	100.02	3.0^*^	−1.67
	Cues to action	Retweets	142.04	86.28	1,681.00^***^	−5.94
		Likes	131.96	89.73	2,195.00^***^	–−4.50
South Korea	Severity	Retweets	174.40	98.61	118.00^**^	–−2.89
		Likes	171.00	98.69	135.00^**^	−2.76
	Susceptibility	Retweets	144.60	98.18	509.00^*^	−2.47
		Likes	141.30	98.35	542.00^*^	–−2.29
India	Cues to action	Retweets	126.18	85.74	2,761.00^***^	−4.76
		Likes	122.71	87.74	3,014.50^***^	−4.11

* *p* < 0.05;

** *p* < 0.01;

*** *p* < 0.001.

To sum up, the most effective HBM construct was cues to action which was effective in inducing Twitter engagement for four countries (the U.S., Germany, Japan, and India). Severity and susceptibility were both effective for South Korea, while severity was partially effective for the U.K. and susceptibility was effective for the U.S. in inducing Twitter engagement. Benefit was effective in inducing Twitter engagement for the U.S. and Japan. Self-efficacy was only effective in inducing Twitter engagement for the U.S. As only four tweets mentioned barriers (one in the U.K. and three in Germany), there were not enough data to examine the effect of barriers on Twitter engagement.

To explore what sub-themes of the HBM constructs were effective in inducing Twitter engagement for each country, we further ran the sub-themes of HBM constructs and Twitter engagement by country (Table 8). Specifically, the severity of COVID-19 to the general public was positively related to Twitter engagement for the U.K. One sub-theme of susceptibility (susceptibility of COVID-19 to vulnerable groups), two sub-themes of benefits (the benefits of personal measures and physical and social distancing measures for the general public), three sub-themes of cues to action (action on physical and social distancing measures for the general public, personal measures and pharmaceutical interventions), and one sub-theme of self-efficacy (training or guidance on personal measures) were positively related to Twitter engagement for the U.S. Cues to action on personal measures were the only positive predictor of Twitter engagement for Germany. The benefits of personal measures and cues to action on personal measures were positively related to Twitter engagement for Japan. The susceptibility of COVID-19 to the general public and the severity of COVID-19 to vulnerable groups were two positive predictors of Twitter engagement for South Korea. Finally, cues to action on physical and social distancing measures for the general public and cues to action on personal measures were two positive predictors of Twitter engagement for India.

**Table 8:** Sub-themes of HBM constructs and Twitter engagement by country.

**Country**	**Sub-theme of HBM constructs**	**Engagement variables**	**Mean ranks of the group with the HBM variable present**	**Mean ranks of the group with the HBM variable absent**	**Mann-Whitney *U***	***Z***
UK	2.2. The severity of COVID-19 to the general public	Retweets	179.50	99.70	40.00^*^	−1.94
		Likes	180.50	99.69	38.00^*^	−1.97
US	1.1. The susceptibility of COVID-19 to the vulnerable groups	Retweets	149.92	98.97	285.50^*^	−2.12
		Likes	145.67	99.10	311.00	−1.94
	3.1. Benefits of physical and social distancing measures for the general public	Retweets	156.00	98.78	249.00^*^	−2.39
		Likes	164.58	98.52	197.50^**^	−2.75
	3.2. Benefits of personal measures	Retweets	141.50	97.18	772.50^**^	−2.85
		Likes	142.13	97.12	763.00^**^	−2.90
	5.3. Cues to action on physical and social distancing measures for the general public	Retweets	131.60	94.13	1,764.50^***^	−3.44
		Likes	134.31	93.58	1,672.50^***^	−3.74
	5.4. Cues to action on personal measures	Retweets	139.30	87.91	1,798.50^***^	−5.40
		Likes	142.36	86.92	1,648.50^***^	−5.83
	5.8. Cues to action on pharmaceutical interventions	Retweets	69.13	103.04	917.00^*^	−2.18
		Likes	84.43	101.80	1,146.50	−1.12
	6.2. Self-efficacy (training or guidance on personal measures)	Retweets	169.00	97.27	243.00^***^	−3.63
		Likes	167.78	97.33	254.00^***^	−3.57
Germany	5.4. Cues to action on personal measures	Retweets	132.43	95.08	1,553.50^**^	−3.21
		Likes	125.76	96.22	1,747.00^*^	−2.54
Japan	3.2. Benefits of personal measures	Retweets	198.00	100.01	2.00^*^	−1.69
		Likes	197.00	100.02	3.00^*^	−1.67
	5.4. Cues to action on personal measures	Retweets	147.19	96.44	725.00^***^	−3.36
		Likes	148.03	96.37	711.50^***^	−3.43
South Korea	1.2. The susceptibility of COVID-19 to the general public	Retweets	142.11	98.54	485.00^*^	−2.21
		Likes	137.67	98.75	525.00^*^	−1.97
	2.1. The severity of COVID-19 to the vulnerable groups	Retweets	189.50	99.60	20.00^*^	−2.19
		Likes	189.50	99.60	20.00^*^	−2.19
India	5.3. Cues to action on physical and social distancing measures for the general public	Retweets	143.50	91.69	1,360.00^***^	−4.76
		Likes	134.74	93.49	1,658.00^***^	−3.79
	5.4. Cues to action on personal measures	Retweets	139.08	90.86	1,657.00^***^	−4.71
		Likes	132.64	92.47	1,914.50^***^	−3.93

* *p* < 0.05;

** *p* < 0.01;

*** *p* < 0.001.

## Discussion

This study aimed to explore to what extent national health departments applied the HBM constructs in their COVID-19-related tweets and the effect of the HBM constructs in messages on Twitter engagement for six national health departments. After comparing the results across six nations, we found, regardless of political and cultural differences, health departments across nations all used the HBM constructs as communication strategies to promote their health policies against COVID-19 on Twitter. Overall, the most often used HBM constructs by the six countries' health departments was cues to action, followed by susceptibility, benefits, self-efficacy, severity, and barriers. This finding is consistent with previous studies that found the HBM constructs were used by national health departments' tweets across nations (14). One thing in common across nations is that the health departments were keen on providing directions (cues to actions) for the public, guiding them on what to do against COVID-19. Different from previous studies that only treated HBM as six single constructs, we also analyzed the sub-themes within each construct. Results showed that actions cued by national health departments varied from movement restrictions, social distancing measures, taking personal measures (e.g., washing hands), protecting vulnerable groups, enhancing medical resources strategies, doing virus testing and patient tracking, to pharmaceutical interventions (see Table 3). Among all the health measures, social distancing measures and personal measures were the most promoted measures across nations.

The question is: did people accept health departments' suggestions? What kind of justifications could convince people to accept those health measures? Based on the data of HBM, we systematically characterized the people of six countries regarding their different responses to their health department's health promotion of COVID-19 on Twitter. We named them seekers of diagnosis, seekers of directions, and seekers of justifications, respectively. The seekers of diagnosis were the people who were still seeking the nature (severity and susceptibility) of COVID-19, while the seekers of directions and justifications were keen on knowing “what to do” (cues to actions) and why to do (benefits, barriers, self-efficacy), respectively.

### South Korean and the U.K. people as *seekers of diagnosis*

First, we named the people of South Korea and the United Kingdom as “seekers of diagnosis.” The public of these two nations positively reacted to those tweets that mentioned the severity or susceptibility of the epidemic, while not positively responding to the tweets that mentioned the justification or directions for actions. HBM theorists argued that the constructs of severity and susceptibility not only refer to the knowledge of a disease but also the necessity of further health measures (5). Borrow their insights, in our study, we regard these two constructs as a *diagnosis* that legitimize or illegitimate further treatments. Without convincing people how serious COVID-19 was and how vulnerable people could be exposed to such disease, it is impossible for the health departments to further persuade people to take action against it. The data indicated that in the first year since the COVID-19 outbreak, the people of the U.K. and South Korea on Twitter were still seeking the nature, rather than the treatments, of COVID-19. Between these two groups of diagnosis seekers, the U.K. people were concerned more about how vulnerable they would be exposed to the epidemic, while the Korean citizens were concerned about both the severity and vulnerability of the disease.

### German and Indian people as seekers of directions

The very opposite of the above two countries was Twitter users from Germany and India. Those only positively responded to those tweets that mentioned “cues to actions”, were named “seekers of directions.” According to HBM, the construct of cues to actions refers to the stimulus cues that direct individuals to implement certain health behaviors (4). People from Germany and India used “like” to express their support and retweeted the relevant tweets to share them with their followers. The data indicated that, rather than focusing on a diagnosis of the disease or a justification of health measures, many German and Indian people have reached a conclusion that a health measure should be taken, and they were actively seeking a clear direction of “what to do” rather than “why to do it.”

Interestingly, though both actively seeking for directions from the health departments against COVID-19, Indian citizens were more willing to support and share those tweets mentioning the directions of personal measures (e.g., washing hands, wearing masks) and social distancing measure, while German people only significantly liked and retweeted the tweets that called for taking personal measures. For health departments of Germany and India, keeping social distancing and taking personal measures were the two most frequently suggested actions (see Table 3), yet German people selectively reacted to the personal measures only. Our analysis illustrates that people's positive reactions to cues to actions did not mean that they supported all the health measures their health departments call for. Researchers need to pay attention to people's selective acceptance (or rejection) of certain health measures.

### The U.S. and Japanese people as *seekers of justifications*

We named the last two groups, the Twitter users from the U.S. and Japan, as seekers of justifications. On the one hand, like German and Indian people, the U.S. and Japanese people positively reacted to the construct of cues to actions (directions); on the other hand, they were also seeking justifications for such directions during the first year since the COVID-19 outbreak. In the case of the U.S., people were likely to share and like the tweets that mentioned cues to actions of social distancing measures, taking personal measures, and pharmaceutical interventions. Meanwhile, they positively reacted to those tweets that explained the necessity of those actions, from “the possibility of infection with COVID-19” (susceptibility), “the benefits of health measures” (benefits), to “training or guidance on how the health measures can be successfully implemented” (self-efficacy). People in Japan positively reacted to the tweets that urged them to take personal measures against COVID-19, and those tweets that mentioned the benefits of the health measures.

In sum, we used HBM to characterize sampled people in six different countries, based on their reactions to the health promotion tweets posted by the health department in each country. We found that overall speaking, the people in Germany, India, the U.S., and Japan were more on the “convinced side” since they positively reacted to those clear directions of “what to do against COVID-19”; Yet the U.S. and Japan were also eager to know the justifications of such directions, to know “why to do it.” People in South Korea and the U.K. were still seeking a diagnosis, instead of health measures, of COVID-19. We also found that people from six countries responded differently to the health measures that health departments suggested. For example, all the health departments promoted a social distancing policy and took personal measures using the construct of cues to actions; however, only people from two countries (the U.S. and India) actively reacted to such measures. It is very clear from the data that the social distancing policy is more controversial and debatable.

This study has several limitations that could be addressed by future research. First, to make the countries comparable, we drew our sample from the six countries with the highest GDPs. Countries with low GDPs were overlooked in our study. Future studies could compare the difference between countries high in GDP and those with low GDP to see whether the HBM constructs also apply to countries low in GDP. Second, the study focused on those who would consider the views of the health departments. Those who distrusted health departments would not have even followed their official Twitter accounts or paid attention to the departments' suggestions. This is the missing piece of the puzzle of the current study, and we call for future studies to consider how to include those people in the research. Third, this study only sampled 1,200 tweets from health departments in six countries. Future research can expand the sample size of each country and include more countries in the analysis.

## Conclusion

HBM was once regarded as an “outdated” theory (22), an old-fashioned behavioral model that predicts health behaviors in surveys. However, this study proved that HBM worked well in the digital media era, which can sketch the health measures promoted by the policymakers, evaluate the *specific* health promotion strategies that the policymakers use to promote the health measures and profile the people exposed to the health promotion. This study also broadened the use of HBM, providing a comprehensive framework for future big data research to examine health promotion.

## Implications for policymakers

Policymakers can implement HMB to understand the public and review the effectiveness of their promotion strategies, knowing the needs of their people more efficiently. Based on HBM, policymakers can quickly locate the needs (those HBM constructs that are mostly liked and retweeted) and doubts (those constructs that are ignored and less liked) of the citizens and rebuild their promotion strategies swiftly. We call for policymakers to pay specific attention to the “gap” between the health measures they have released and the echoes from the public, finding out the health measures that are not positively received by their people. In this way, policymakers can further improve their health promotion strategies.

## Data availability statement

The raw data supporting the conclusions of this article will be made available by the authors, without undue reservation.

## Author contributions

ZM and DW conceived the idea of the research, collected and analyzed the data, and prepared the first and subsequent drafts. SZ participated in data collection and analysis. All authors contributed to the article and approved the submitted version.
